# Identification of Appropriate Reference Genes for Normalizing miRNA Expression in Citrus Infected by *Xanthomonas citri* subsp. *citri*

**DOI:** 10.3390/genes11010017

**Published:** 2019-12-23

**Authors:** Shiheng Lyu, Ying Yu, Shirong Xu, Weiwei Cai, Guixin Chen, Jianjun Chen, Dongming Pan, Wenqin She

**Affiliations:** 1 College of Horticulture, Fujian Agriculture and Forestry University, Fuijan 350002, China; 2140305002@fafu.edu.cn (S.L.); 2017204032@njau.edu.cn (Y.Y.); 2170305004@fafu.edu.cn (S.X.); 1170371005@fafu.edu.cn (G.C.); 2 Department of Environmental Horticulture and Mid-Florida Research and Education Center, Institute of Food and Agricultural Sciences, University of Florida, Apopka, FL 32703, USA; 3 College of Horticulture, Nanjing Agriculture University, Nanjing 210095, China; 4 College of Crop Science, Fujian Agriculture and Forestry University, Fuzhou 350002, China; 2170101002@fafu.edu.cn

**Keywords:** citrus canker, *Citrus sinensis*, *Citrus reticulata*, microRNA, Poly(A) RT-qPCR, reference gene, stem-loop RT-qPCR, *Xanthomonas citri* subsp. *citri*

## Abstract

MicroRNAs (miRNAs) are short noncoding RNA molecules that regulate gene expression at the posttranscriptional level. Reverse transcription-quantitative PCR (RT-qPCR) is one of the most common methods used for quantification of miRNA expression, and the levels of expression are normalized by comparing with reference genes. Thus, the selection of reference genes is critically important for accurate quantification. The present study was intended to identify appropriate miRNA reference genes for normalizing the level of miRNA expression in *Citrus sinensis* L. Osbeck and *Citrus reticulata* Blanco infected by *Xanthomonas citri* subsp. *citri*, which caused citrus canker disease. Five algorithms (Delta Ct, geNorm, NormFinder, BestKeeper and RefFinder) were used for screening reference genes, and two quantification approaches, poly(A) extension RT-qPCR and stem-loop RT-qPCR, were used to determine the most appropriate method for detecting expression patterns of miRNA. An overall comprehensive ranking output derived from the multi-algorithms showed that poly(A)-tailed miR162-3p/miR472 were the best reference gene combination for miRNA RT-qPCR normalization in citrus canker research. Candidate reference gene expression profiles determined by poly(A) RT-qPCR were more consistent in the two citrus species. To the best of our knowledge, this is the first systematic comparison of two miRNA quantification methods for evaluating reference genes. These results highlight the importance of rigorously assessing candidate reference genes and clarify some contradictory results in miRNA research on citrus.

## 1. Introduction

Citrus is one of the most important fruit crops due to its high nutritional and economic value. Citrus crops are cultivated in a wide range of regions with a total fruit production over 130 million tons in 2015, ranking first in quantity in world fruit production (FAO, 2017). Citrus production, however, has encountered several serious diseases, including citrus canker and Huanglongbing [1]. Citrus canker is caused by *X. citri* subsp. *citri* (*Xcc*) with symptoms of water-soaked eruptions, circular lesions, and pustule-like lesions on all plant tissues, resulting in heavy economic losses [2,3]. 

Research into citrus canker has been focused on the screening of genetic resources for disease resistance [4,5], transcriptomic analysis of plant responses to pathogen infection [6,7,8], induced resistance for disease control [3], and genome editing of specific genes [9]. Plant defense response is comprised of complex molecular networks regulating gene expression at both transcriptional and posttranscriptional levels [10]. MicroRNAs (miRNAs) are a class of non-coding small RNA (sRNA) of unequal lengths ranging from 20 to 25 nt, that have emerged as ubiquitous posttranscriptional regulatory molecules and participate in extremely important biological functions in both plants and animals [11,12]. 

In plants, miRNAs are known to regulate plant–pathogen interaction by sRNA-mediated gene silencing pathways [13]. Thus far, no mechanistic analysis has been conducted on posttranscriptional regulation of citrus under *Xcc* challenge. miRNA microarray and sRNA sequencing have been used to investigate miRNA-profiling [14]. High-throughput sRNA sequencing and microarray data from a large number of disease samples provide important information on miRNA expression [15,16,17]. To determine the expression level of mature miRNAs of interest, the reverse transcription-quantitative polymerase chain reaction (RT-qPCR) is commonly employed for the quantification due to its high sensitivity, good reproducibility and superior operability [18]. The level of the expression is normalized by comparing with reference genes [15,18]. Therefore, the selection of reference genes is critically important for the accuracy. Candidate reference genes could be selected from the output of high-throughput sRNA sequencing. These candidate genes were further evaluated by RT-qPCR for their stability, and those with least variability are chosen for miRNA analysis [19,20,21]. 

The earliest experiment on quantifying target gene expression using RT-qPCR in citrus was performed in 2010. Analysis through geNorm and NormFinder indicated that *EF1a* and *ADP* were the most stable reference genes in “Swingle” citrumelo under drought stress [22]. Two other genes *FBOX* and *GAPDH* were considered to be appropriate reference genes in citrus genotypes infected by five pathogens (*Alternaria alternata, Phytophthora parasitica, Xylella fastidiosa, and Candidatus Liberibacter asiaticus*) [23]. For determining gene expression in different organs and tissues, *CitUBQ14*, *CitUBL5,* and *CitGAPDH* appeared to be stably expressed in citrus [24,25]. These reference genes are valuable for RT-qPCR analysis in citrus research. However, they are protein-coding housekeeping genes and may not be appropriate for the analysis of miRNA expression [21]. Firstly, the population of miRNA is much smaller than that of mRNA, with the numbers of expressed molecules in the hundreds rather than tens of thousands, and thus more susceptible to distortion from trends [21]. Secondly, miRNAs are largely posttranscriptionally regulated, and therefore their mature expression may not behave with a high degree of independence from many of their fellow miRNAs [26,27]. Moreover, increasing evidence suggests that global shifts in miRNA expression can be caused by physiological and pathological reasons or by induction [21]. These characteristics make the normalization of miRNA expression significantly different from that of mRNA expression. Therefore, research into the identification of appropriate reference genes for quantifying miRNA expression becomes increasingly important. 

Conventional PCR analysis is not suitable for short RNA targets averaging 22 nt in length, because mismatch from closely related miRNAs, precursors and genomic sequences exert an effect on the accuracy due to the low specificity and sensitivity. Currently, two complementary DNA (cDNA) synthesis methods for miRNA quantitative analysis (stem–loop reverse transcription and poly-A tail extension) have been used for compensating these deficiencies [28,29]. Stem–loop RT requires that the primer contains a self-complimentary sequence and a designed overhang. This overhang binds to the 3’ portion of the mature miRNA sequence and can be reverse transcribed with MMLV reverse transcriptase. The mature miRNA sequence will degrade by denaturation, resulting in a complimentary copy of the miRNA forms the template [26]. Poly A tail extension was very close to conventional Oligo(dt) primed reverse transcription reaction, where total RNAs, including mature miRNAs, undergo polyadenylation by using *Escherichia coli* poly(A) polymerase. Poly(A)-tailed miRNA can be converted into cDNA along with mRNAs in a reverse transcription reaction primed by a standard poly(T) anchor adaptor and MMLV reverse transcriptase [29].

Several studies have recently been performed for identifying appropriate reference genes for miRNA qPCR, of which the stem–loop method has been used more frequently. A set of miRNAs was selected for evaluation in plants, such as castor bean, cotton, watermelon, wheat and sugarcane [30,31,32,33]. Luo et al., 2014, found miR5059 and miR5072 were the best reference genes in different peach tissues under different abiotic stress treatments using poly(A)-tailed RT-qPCR approach [34]. In *Arabidopsis*, stress-induced miRNAs were cloned by sequencing and the computational method [35,36]. Due to their extremely high expression, U6 snRNA and 5S rRNA/tRNA were used as reference genes to detect the expression of *Arabidopsis* miRNAs in blot assays [37]. tRNA and 5S rRNA were also applied to monocotyledon plants [38]. More novel stress-responsive miRNAs were reported in *Populus trichocarpa* [39], which are absent in *Arabidopsis*. The 5.8S rRNA was selected as a reference gene to quantify miRNAs by using stem–loop real time PCR [39]. Additionally, actin as a protein-coding gene, is widely used in RT-qPCR due to its stable and high expression in different cells. A wheat actin gene was selected to normalize the quantity of templates added in the PCR reactions for analyzing the expression of novel miRNAs using semi-quantitative RT-PCR [40]. Actin was also used as an endogenous control gene to normalize the expression of miRNAs in *C. sinensis* [41,42,43,44] and in tomato after inoculation with *Phytophthora infestans* [45]. Other stably expressed protein-coding genes, such as *GAPDH* or *EF-1a*, are also used as internal standards in miRNA quantitative studies [46,47]. Due to the specific characteristics of miRNA mentioned above, the use of protein-coding genes as references for normalizing miRNA expression could be questionable. 

To our knowledge, there has been no study on the identification of appropriate reference genes for quantifying miRNA expression using poly(A) extension RT-qPCR and stem-loop RT-qPCR in plants. This study was intended to address this issue using *C. sinensis* (L.) Osbeck and *Citrus reticulata* Blanco infected with the citrus canker pathogen. To normalize citrus miRNA expression, we first evaluated the expression stability of 20 candidate reference genes including common protein-coding genes. We then used the stem–loop method and poly(A)-tail extension to evaluate the expression variation of candidate miRNA in two citrus species under *Xcc* treatments. Our results showed that candidate reference gene expression profiles determined by poly(A) RT-qPCR were more consistent in the two citrus species.

## 2. Materials and Methods 

### 2.1. Plant Materials, Growth Conditions, and Bacterial Inoculation

Two citrus species differing in the susceptibility to *Xcc* were used in this study. Sweet orange, *C. sinensis*, is moderately susceptible, whereas Ponkan mandarin, *C. reticulata*, is moderately resistant [48]. Seeds of sweet oranges ‘Xuegan’ and Ponkan mandarin were collected from Fuzhou, Fujian, China. After sprouting, they were planted in black plastic pots (12 × 12 cm) filled with a substrate comprised of 50% peat, 20% pine bark, 20% perlite and 10% coarse sand. Seedlings were placed in a greenhouse at 30/25 °C (day/night) and 70% of relative humidity under natural light.

*Xcc* was cultured in the NB (nutrient broth) medium with 1.5% agar at 28 °C. Bacterial cells were suspended in sterile water. The suspension at 1 × 10^5^ cfu mL^−1^ was syringe-infiltrated into the young leaves of four plants per species. Injection of sterile water to four plants of respective species was a control treatment. Leaf discs were taken from each plant on days 0, 1, 2 and 4 post-infiltration, frozen in liquid N_2_, and stored at −80 °C until RNA extraction. Thus, there were four biological samples (replicates) per species on a given day. 

### 2.2. RNA Isolation and Complementary DNA Synthesis

Total RNA was extracted from collected discs taken on days 0, 1, 2 and 4, respectively, using Trizol reagent (Invitrogen, Carlsbad, CA, USA) following the instruction from the manufacturer. RNA concentrations and ratios of absorbance at 260 nm to that at 280 nm (260/280) or 230 nm (260/230) were determined using a Nanodrop 1000 spectrophotometer (Thermo, Wilmington, DE, USA).

For the use of the poly-A tail method, each polyadenylation and reverse transcription reaction was performed with 1 μg of total RNA in a final volume of 10 μL using a Mir-X™ miRNA First Strand Synthesis kit (Takara/clontech, Mountain View, CA, USA) where there were 5 μL mRQ Buffer (2×) and 1.25 μL mRQ Enzyme. The reaction tubes were incubated in a thermocycler for 1 h at 37 °C and terminated at 85 °C for 5 min to inactivate the enzymes.

For the stem–loop reverse transcription method, the experimental RNA (up to 1 μg), 1 μL (1 μM) stem–loop specific primer (Table S1), and 1 μL (50 μM) Oligo(dT) 15 primer (Promega, Madison, WI, USA) were mixed in nuclease-free water for a final volume of 5 μL per RT reaction and incubated at 70 °C for 5 min followed by ice-cooling. cDNA synthesis was carried out using Improm-II reverse transcription system according to the manufacturer’s instruction (Promega). Briefly, the reaction thermal profile was set with an initial temperature of 16 °C for 30 min followed by 60 cycles of 30 °C for 30 s, 42 °C for 30 s and 50 °C for 1 s. After 60 cycles, the reaction was terminated by incubating the samples at 85 °C for 5 min [28]. 

### 2.3. Selection of Candidate Reference Genes and Primer Design

Our previous study identified the transcription levels of conserved miRNAs from sweet orange and Ponkan mandarin infected with *Xcc* using the high-throughput sequencing. NEB Next Ultra small RNA Sample Library Prep Kit for Illumina (New England, NEB, USA) was employed for generating all 12 sequencing libraries. The Illumina HiSeq2500 platform was used to sequence the library, thereby generating a total of 260,117,346 raw reads. The transcription levels of 20 conserved miRNAs (including published reliable references miR171 [32], miR166b [31], miR159a [49], miR396a [50] and miR166b [51] were obtained and analyzed. Eight putative reference RNA targets (miR472, miR428b, miR396a, miR166b, miR3954, miR160, miR162-3p and miR403) with a median expression and the smallest standard deviations were selected from high-throughput sequencing data for identifying the most suitable normalizer for miRNA qPCR in citrus infected by *Xcc*. 

For the stem–loop method, the primers (Table S1) for miRNAs cDNA synthesis and forward miRNA primers for qPCR were designed according to the report of Chen et al. (2005) [28] or by using the online tool (http://150.216.56.64/miRNAprimerDesigner.php). Universal reverse primer (Table 1) was also based on the report of Chen et al. (2005) [28]. For Poly-A tail primer design, miRNA specific 5’ primer was used as the entire sequence of mature miRNA (21–23 nt). The 3’ primer for qPCR was the mRQ 3’ Primer supplied with the Mir-X™ miRNA First-Strand Synthesis kit. 

Non-coding RNA (U4, U5, U6 and snoR14) primers (Table 1) were obtained from previous research [52]. Eight protein-coding traditional reference genes (*ACTIN1*, *ACTIN2*, *UBC28*, *TUA5*, *EF1α*, *TUB4*, *GAPDH* and *PP2A*) reported to be good potential candidates in previously published studies [53,54,55,56] also included in this study. The sequences of citrus protein-coding traditional candidate reference genes and genes coding for the *CsLOB1* transcription factor were extracted from citrus genome data (http://citrus.hzau.edu.cn/orange/index.php). Primer design was made according to a standard set of design criteria (e.g., primer Tm, length and GC content), which generated a unique, short PCR product of the expected length and sequence [57]. 

### 2.4. Reverse Transcription-Quantitative PCR Analysis

The reaction mixture with a total volume of 20 μL contained 2 μL diluted cDNA, 0.4 μL of each 10 μM primer, 7.2 μL ddH_2_O and 10 μL SYBR Advantage Premix (Takara). Four biological replicates for each sample together with two technical replicates for each well were performed. The RT-qPCR conditions were as follows: denaturation at 95 °C for 10 s, followed by 40 cycles of denaturation at 95 °C for 5 s, and annealing at 60 °C for 30 s in CFX96 Touch™ Real-Time PCR detection system (Bio-Rad, Hercules, CA, USA). After amplification, a melting curve analysis (60–95 °C with at increments of 0.5 °C) was generated for each reaction to verify the specific amplification.

### 2.5. Data Analysis

A serial of 5-fold dilutions of cDNA (5×, 25×, 125×, 625× and 3125×) from sweet orange pooled samples were used to create standard curves. The amplification efficiencies (E%) and the correlation coefficient (*r*) for all primer pairs were determined by RT-qPCR standard curve assays. The PCR efficiency was calculated with following the formula E = 10^−1^/slope −1. All reference gene’s stability was evaluated using free algorithms: the Delta-Ct method [58], geNorm [59], NormFinder [60] and BestKeeper [61]. The comprehensive stability analysis was performed by a web-based tool RefFinder (http://150.216.56.64/index.php) [62]. 

The four common statistical approaches (Delta-Ct method, geNorm, NormFinder and BestKeeper) might yield contradictory results from the same data. However, RefFinder could make comprehensive stability ranking of candidate genes according to the results from these softwares. Eight miRNA genes were analyzed in a comparative way to determine the most reliable normalizer, data from stem-loop RT and poly(A) extension were imported to RefFinder individually. To evaluate the potentiality of these candidate genes as miRNA reference gene, we also analyzed their expressional stability by combining data from protein coding RNA.

### 2.6. Reference Gene Validation

A citrus canker-induced gene Lateral Organ Boundaries (*CsLOB1*) was analyzed in *C. sinensis* by RT-qPCR [63], *CsLOB1* primers was designed according to the previously mentioned methods, and the same conditions and criteria as for the reference genes were used in *CsLOB1*. To calculate the relative fold changes in the gene expression of sweet orange leaves after *Xcc* inoculation, the relative expression of the target was calculated using the comparative 2^−ΔΔ^*C*t method and normalized to the selected reference genes.

## 3. Results

### 3.1. Verification of Primer Efficiency for the Candidate Reference Genes

The correlation coefficient (*r*) represents whether the plotted data points fit the regression line generated by the standard curve. As a good linear performance, *r* should be more than 0.99. In this study, *r* of the standard curve ranged from 0.9730 to 0.9998 (Table 1). The amplification efficiency (E) refers to amplification rate of a template during the reaction. The theoretically ideal E value is 100% indicating that the target cDNA template duplicates exponentially in every PCR cycle [64]. The amplification efficiency of the stem–loop primers varied from 86.8% to 104.7%, and the poly(A)-based reaction E value ranged from 91.5% to 103.6% (Table 1). In practical terms, primer efficiency between 90% and 110% was thought to be acceptable for qPCR experiments [65]; thus, all the primer pairs were deemed sufficiently designed. All of the non-coding RNAs showed low amplification efficiency, which was not fit for qPCR experiment.

### 3.2. Ct Value Distribution of Candidate Reference Genes

**Figure 1:**
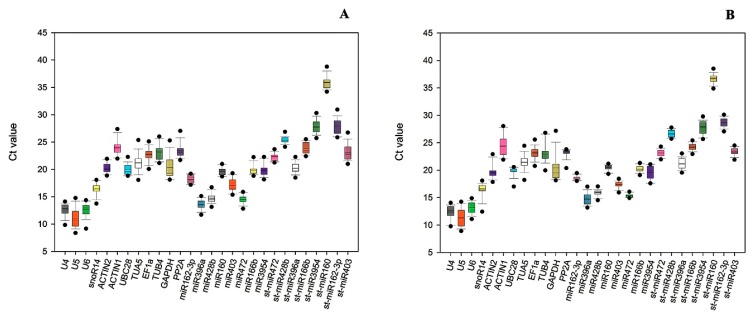
The cycle threshold (Ct) variation of individual candidate reference gene. The Box-whisker plot shows Ct variation of 18 candiates in (**A**) sweet orange and (**B**) Ponkan. A line across the box depicts the median. In each box, the upper and lower edges indicate the 25th and 75th percentiles. Whisker caps are the minimum and maximum values.

The cycle threshold (Ct) values of most genes ranged from 15 to 30, the non-coding RNA (U4, U5 and U6) showed relatively high expression levels in both sweet orange and Ponkan, with the Ct values lower than 15 (Figure 1). St-miR160 had a highest Ct value of 36.87, which was not recommended (15 ≤ Ct ≤ 30) for a RT-qPCR [66]. St-miR472 showed the narrowest Ct ranges (21.61 to 23.75) in sweet orange (Figure 1A), whereas the non-coding RNA gene U5 was the most variable one. Moreover, the Ct value of the stem–loop primer was more variable than poly(A) extension microRNA in all the tested samples. The variability represents a difference between max Ct and min Ct. St-miR162-3p and St-miR403 showed high variability in sweet orange, with their Ct values ranging from 25.83 to 31.31 and 21.61 to 26.97, respectively (Figure 1A). Similarly, St-miR160 showed the highest Ct value in Ponkan (Figure 1B), while Ct variance of miR162-3p and miR472 was minimal. In Ponkan plants where the Ct value ranged from 15 to 20, the expression was relatively stable. 

### 3.3. Reference Gene Stability Analyzed by geNorm, NormFinder, BestKeeper, Delta-Ct Method, and Refinder

To analyze the expression stability of candidate reference genes during different infection stages of citrus leaves, four statistical algorithms (Delta-Ct method, BestKeeper, NormFinder, and geNorm) and a web-based tool (RefFinder) were used to rank the stability of 28 reference genes including 8 miRNA genes reverse transcribed by stem-loop method (Table 2).

All the Ct raw data were directly input to the program BestKeeper. The Ct value of genes with standard deviation (SD) < 1 was considered to be stable enough or SD (± x-fold) < 2 was also a statistical parameter to determine the reference genes. A matrix of pairwise comparisons and *r* calculation was performed using BestKeeper. Genes with higher expression stability should have a *r* closer to 1. Based on the BestKeeper analysis, *U5*, *TUA5*, *GAPDH* and st-miR162-3p could not pass the filter with SD values of 1.44, 1.17, 1.74 and 1.12, respectively, which were much higher than 1 in sweet orange (Table 3). On the other hand, st-miR472 and miR160 (SD: 0.42 and 0.48) were confirmed to be the most stably expressed. Meanwhile, the BestKeeper calculation also provided two best reference genes miR162-3p and miR472 with low SD values (0.25 and 0.26). *GAPDH* was the least stably-expressed gene for the two citrus species with *r* values over 25 (Table 3 and Table 4).

**Figure 2:**
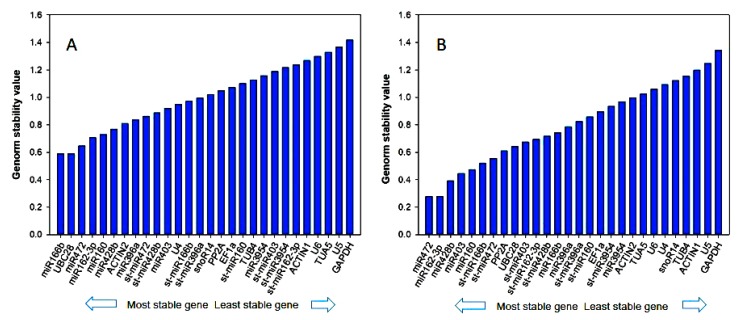
Expression stability analysis of candidate reference genes in (**A**) sweet orange and (**B**) Ponkan calculated by statistical program geNorm. The most stable genes are on the left and the least stable genes on the right.

The geNorm is a popular algorithm to determine the most stable reference genes from a set of tested candidate reference genes in a given sample panel. We identified miR166b, UBC28, miR472 and miR162-3p as the most stable genes in sweet orange according to the average expression stability value *M* using geNorm software (Figure 2A). In geNorm, the lower *M* value represents the higher stability. It is noteworthy to note that all the reference genes in sweet orange were deemed unstable under citrus canker infection according to the commonly proposed cutoff value of *M* ≤ 0.5. Using a *M* threshold value of ≤ 0.5, miR472, miR162-3p, miR427b, miR403 and miR160 had the lower *M* value (0.2790, 0.2790, 0.3930, 0.4450 and 0.4720, respectively) indicating these genes were the most stably expressed genes in Ponkan under canker infection (Figure 2B). The protein coding gene *GAPDH* was the least stably expressed gene in two citrus species.

**Figure 3:**
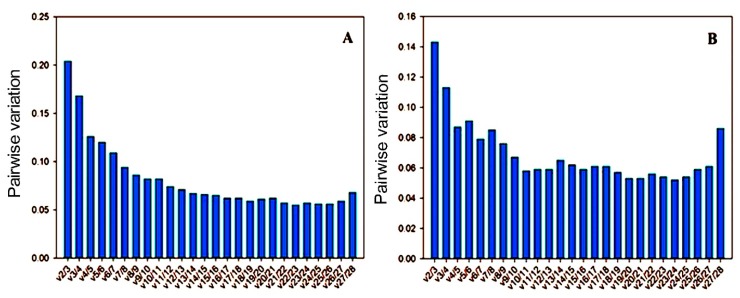
Pairwise variation (Vn/Vn+1) analysis of the candidate reference genes in (**A**) sweet orange and (**B**) Ponkan. The pairwise variation (Vn/Vn+1) was analyzed based on geNorm algorithm to determine the optimal number of reference genes for accurate normalization. We proposed 0.15 as a threshold value, which suggested that adding one more gene into the combination of reference genes is not required.

Additionally, geNorm is also based on a pairwise comparison model to determine the optimal number of reference genes for accurate data normalization. Average pairwise variation Vn/n + 1 with a cut-off score of 0.15 determined the minimum number of genes necessary for data normalization, below which the inclusion of an additional reference gene had no significant contribution to the normalization. For reference genes expression in sweet orange, the analysis revealed that the V3/4 value was 0.168, the normalization factor should preferably contain at least the four best reference genes. While the V4/5 value was 0.126 (Figure 3A), the fifth additional gene was not necessarily needed to normalize the expression, which confirmed the geNorm results. In all of the Ponkan sample sets, the V2/3 value was considerably smaller than the default threshold value of 0.15, suggesting that the two most stable reference genes were miR162-3p and miR472, which should be appropriate for normalization (Figure 3B). 

**Figure 4:**
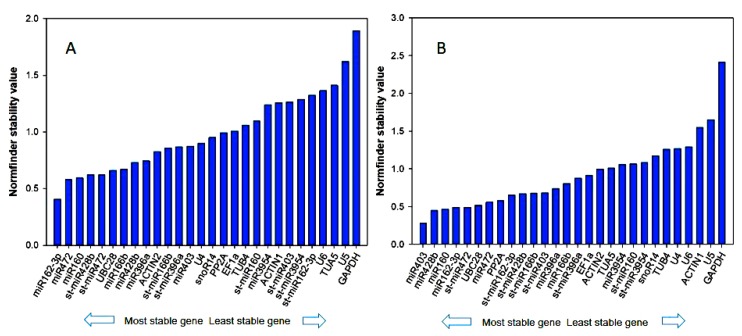
Expression stability analysis of candidate reference genes in (**A**) sweet orange and (**B**) Ponkan calculated by statistical program NormFinder. The most stable genes are on the left and the least stable genes on the right.

We further used NormFinder for identifying appropriate reference genes. NormFinder is a Visual Basic Application for Excel. Like geNorm, the software calculates the expression stability value (M) for all candidate genes. Using an analysis of variance (ANOVA)-based comparison model, NormFinder can estimate the variation of the intra- and inter-group. Based on these two statistical methods, the lowest *M* always suggests the most reliable pair of reference genes. In the sweet orange sample set, the NormFinder analysis revealed that the most stably expressed genes were miR162-3p and miR472, followed by miR160 (Figure 4A). Furthermore, the analysis indicated *GAPDH* as the least stably expressed gene. The algorithm also selected an optimal pair of reference genes in Ponkan, and the most stable ones were miR403 and miR428b with an *M* value of 0.28 and 0.45 (Figure 4B). 

**Figure 5:**
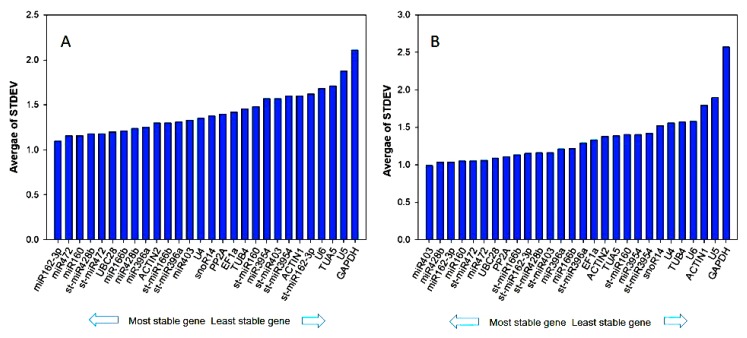
Expression stability analysis of candidate reference genes in (**A**) sweet orange and (**B**) Ponkan calculated by Delta Ct method. The most stable genes are on the left and the least stable genes on the right.

To obtain the most suitable reference genes, we evaluated the candidates by the Delta Ct method. This approach was employed by comparing the mean difference of ‘gene pairs’ and the variation in Ct values within each sample. The ranking of the reference genes from this method was similar to that obtained by the NormFinder algorithm. Delta Ct method analysis found that miR162-3p and miR472 had lowest StdDev, followed by miR160 which suggested that miR162-3p and miR472 were two top-ranked, stable reference genes among all of the studied genes in sweet orange (Figure 5A), which also confirmed the results from NormFinder analysis. For Ponkan (Figure 5B), miR403 followed by miR482b and miR162-3p, were found to be the most stably expressed genes with low StdDev (0.09, 1.04 and 1.04 respectively).

**Figure 6:**
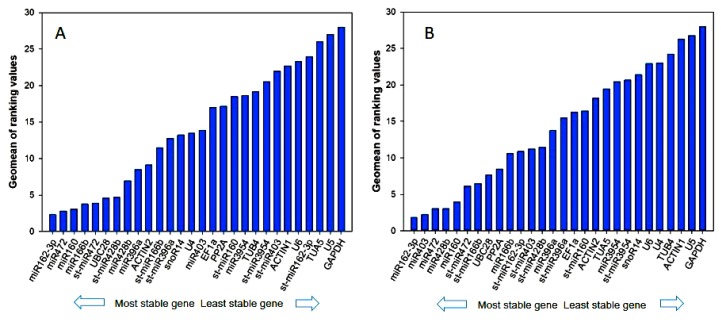
Expression stability analysis of candidate reference genes in (**A**) sweet orange and (**B**) Ponkan calculated by the statistical program RefFinder. The most stable genes are on the left, and the least stable genes on the right.

RefFinder is a web-based tool integrating the above four computational statistical approaches to produce a comprehensive evaluation based on the geometric mean for each candidate gene. In the end, all untransformed raw data values were imported to RefFinder, then the software output ranks reference genes according to the expression of reliability. When the stabilities from all the samples were combined, the output results agreed with the results of NormFinder software and Delta Ct methods. An overall comprehensive ranking output revealed that miR162-3p (Figure 6A), followed by miR472, miR160 and miR166b (Geomean: 2.3, 2.78, 3.08, and 3.74, respectively) were the most stably expressed genes in canker-infected sweet orange, while *U5* and *GAPDH* were the least stably expressed. The RefFinder algorithm showed that miR162-3p and miR403 constitute the best combination of two genes with comprehensive ranking values of 1.86 and 2.21 in all of the Ponkan samples (Figure 6B). Again, *U5* and *GAPDH* were ranked as among the least stably expressed genes, which had the highest Geomean.

The top three most stable genes were miR162-3p, miR160 and miR472 for the poly(A) extension method in sweet orange samples (Table 5). For the stem–loop method, st-miR396a, st-miR162-3p and st-miR166b were ranked as among the most stable genes, while st-miR3954 was the least stable genes under stress conditions. According to RefFinder, the comprehensive ranking poly(A) tail miRNA from the most stable to the least stable gene for Ponkan samples was as follows: miR160 > miR162-3p > miR472 > miR403 > miR428b > miR166b > miR396a > miR3954. While the comprehensive ranking of stem-loop miRNA for Ponkan samples was as follows: st-miR403 > st-miR162-3p > st-miR166b > st-miR160 > st-miR428b > st-miR396a > st-miR3954.

Taken together, *UBC28*, *PP2A* and *EF1a* were identified to be the most stable protein coding reference genes in all sweet orange samples. The protein coding reference genes for accurate transcript normalization in Ponkan were similar to those of sweet orange.

### 3.4. Validation of Candidate Reference Genes

**Figure 7:**
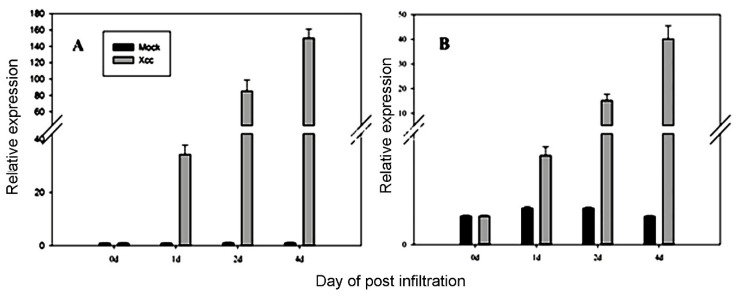
Relative expression of C*sLOB1* in *C. sinensis* after *Xcc* infection or mock using the selected candidate reference genes: miR162-3p and miR160 (**A**) and *GAPDH* (**B**) for normalization. Means ± standard deviation (SE) were calculated from four discs per treatment (*n* = 4) per given day.

To confirm the stability of the best ranked candidate reference genes, the expression pattern of *CsLOB1* was examined in response to *Xcc.* The results clearly indicated that *CsLOB1* was significantly upregulated after *Xcc* infection using the combination of miR162-3p/miR472 for normalization (Figure 7A). Correspondingly, there was little difference in mock at each sampling day. However, when the poor reference gene *GAPDH* was used as an internal control, the expression of *CsLOB1* showed a slight fluctuation in mock, but the expression differences between treatment and mock were significantly reduced (Figure 7B).

## 4. Discussion

A large body of evidence has shown that miRNA-mediated posttranscriptional regulation plays a fundamental role in plant responses to stressful factors, including pathogen infection, salt, drought, heat, cold, nutrient deficiency and metal toxicity [81,82,83,84,85,86]. A great deal of effort has been focused on the identification and functional analysis of plant miRNAs in response to the infection of pathogens, such as viruses [87,88,89], bacteria [16,90,91,92] and fungi [13,93,94], which have provided valuable information as to the role miRNAs play in plant–pathogen interactions [95,96].

Northern blot, microarray and high-throughput sequencing have been used to analyze miRNA expression. However, RT-qPCR is still the most commonly used method. In RT-qPCR analysis, reference genes play a vital role in quantifying the expression level of target genes. Even a slight change in miRNA expression may affect targeting mRNAs for cleavage or repressing translation [96]. Improper reference genes may lead to inaccuracy or false results and incorrect conclusions [97,98]. Recently, there is an increased reporting on the evaluation of plant reference genes under different conditions [30,50,99,100]. However, limited information is available on miRNA reference genes in phytopathogenic research. Several studies showed miRNA in citrus plants [44,45,85], but there is no report on appropriate reference genes for normalizing miRNA expression in citrus canker-infected plants.

The present study identified appropriate reference genes for quantifying miRNA expression in two citrus species infected by *Xcc*. To achieve reliable results without bias, we selected 28 likely reference genes, including protein-coding RNA and non-coding RNA, and evaluated their expression levels using two different methods in the two citrus species, differing in resistance to *Xcc*. We then analyzed the performance of the reference genes by five methods and identified appropriate reference genes for each species. miR162-3p, miR160 and miR472 performed best as reference genes for sweet orange plants infected by *Xcc*, and miR160, miR162-3p and miR472 were the most stable genes for Ponkan attached by the same pathogen. To further confirm the reference genes, we used LOB as the target gene from sweet orange to verify their accuracy. Among the three reference genes, miR160 is known to mediate the interaction between auxin and cytokinin by suppressing the levels of ARF transcription factors, which plays a critical role in various aspects of plant development [101,102,103]. DICER-LIKE 1 (DCL1) is the main enzyme processing miRNA precursors into mature miRNAs and incorporated into the AGO1-RISC, which is considered indispensable for plant development [104]. Meanwhile, miR162 maintains a proper level of DCL1 transcripts by targeting DCL1 mRNA for a negative feedback regulation [104,105,106].

A single reference gene has been commonly used to normalize RT-qPCR results [107]. However, accumulating evidence has suggested that a single reference gene tends to show higher expression variability due to different biological factors [108]. Conversely, the use of multiple reference genes improved RT-qPCR statistical veracity [60,108]. In this study, geNorm software suggested that three reference genes are needed for a more accurate normalization for sweet orange infected by *Xcc*. As for Ponkan, two reference genes are found to be sufficient for accurately quantifying target gene expression levels (Figure 3). Ponkan is a canker-resistant genotype and showed minor physiological changes and metabolic disorders than the susceptible sweet orange plant used in this study. All Ponkan samples remained relatively stable during *Xcc* infection processes at different infection stages. Consequently, Ponkan required fewer reference genes for normalizing qPCR results. Nevertheless, our results documented that the use of miR162-3p, miR160 and miR472 should accurately normalize the expression levels of the target gene in both sweet orange and Ponkan during *Xcc* infection.

*GAPDH* was considered to be a stable reference gene for qPCR normalization in different plants. *GAPDH* encodes key enzymes catalyzing the conversion of glyceraldehyde-3-phosphate to 1,3-biphosphoglycerate in the presence of NAD+ and inorganic phosphate in the glycolytic pathway [109,110,111]. Wu et al. (2014) suggested that *GAPDH* was the most reliable reference gene in navel orange (*C. sinensis* L. *Osbeck*) fruit during five different ripening stages [25]. In contrast, our results indicated that *GAPDH* was unstable, and was not an ideal reference gene for gene expression analyses, which was consistent with the reports of the others [112,113,114,115,116], that *GAPDH* was an unstably expressed gene in different varieties of oranges under different conditions. In another study, a total of 22 *GAPDH* genes were identified in wheat. Members of this family were found to notably respond to abiotic stresses [117]. In other words, some of these *GAPDHs* may participate in multiple functions except for metabolism roles in plants, such as involving abiotic stress resistance. Thus, caution is needed to use the *GAPDH* gene as a reference gene in RT-qPCR analysis. 

*CsLOB1* is transcription factor in the family of the Lateral Organ Boundaries Domain gene. All strains of *Xcc* can encode transcription activator-like (TAL) effectors which bind to effector binding elements in the promoter of *CsLOB1* and induce the expression of disease susceptibility genes [63]. In this study, the identified reference genes (miR162-3p and miR472) showed highly reliable for normalizing *CsLOB1*. The expression profile of *CsLOB1* was consistent with two suitable reference gene normalizations, which was also in agreement with the study of Hu [63]. When *GAPDH* was used as an internal control, *CsLOB1* had no significant change in 24h, and mock showed fluctuating patterns of expression (Figure 7). These inaccurate results indicated that *GAPDH* would be unreliable for RT-qPCR analysis in the citrus bacterial canker response. 

Analyzing miRNA expression profiles has inherent difficulties including (1) short nucleotide (18–24 nt); (2) heterogeneous GC content; (3) no sequence feature [e.g., poly(A)]; (4) target sequence interference; and (5) highly homologous within family [18]. Thus, poly(A) extension RT-qPCR and stem–loop RT-qPCR have been used for analyzing miRNA expression. In the stem–loop RT-qPCR, the reverse transcription of individual mature miRNAs uses a specific stem–loop primer. Due to the use of miRNA-specific primers, this approach is highly specific, thus effectively reducing background noise. This method, however, is time consuming and labor intensive. In the poly(A) extension RT-qPCR, miRNAs are first tailed by *E. coli* Poly(A) Polymerase and then reverse transcribed by oligo(dT) with a universal primer-binding sequence. This method is a universal reverse transcription, the product of the reaction contains cDNA from non-coding RNA (miRNA) and mRNA with a universal anchor primer. Compared to stem–loop RT, the Poly(A) extension method can analyze the transcript level of miRNA, miRNA target genes and other related genes with the same background. In the present study, we evaluated the two methods by comparing reference genes’ stability in different citrus samples through two miRNA quantification methods. Our results showed that the average Ct value of poly-A tail qPCR was lower than the stem–loop RT method. 

Moreover, the efficiency of PCR amplification for the stem–loop primer was lower than the poly-A tail, and some of them were even below 90%. Accordingly, the poly-A tail qPCR has higher amplification efficiency and better sensitivity than the stem–loop RT-qPCR. In forensic casework, poly-A tail extension also exhibited apparently more amplification than stem–loop RT for a body fluid identification of miR-451 and miR-205 [118]. In addition, different statistical algorithms revealed that the rank of the stability of the reference genes was in the following order: Poly(A) extension microRNA > stem-loop microRNA > protein coding RNA > non-coding RNA. In the present study, the poly(A) extension method achieved consistent results in two citrus species. Conversely, the top three stable stem–loop microRNAs were different in sweet orange and Ponkan, except st-miR162-3p. A study of *Triticum dicoccoides* showed that the results of stem–loop RT-qPCR were only partially consistent with data obtained from sequencing or microarray [119]. Similar results have been reported in *Triticum aestivum* L., where five miRNA genes in different wheat tissues were analyzed by two miRNA qPCR approaches, as well as deep sequencing. The deep sequencing results had higher correlation coefficients with results obtained from poly(A) RT-qPCR, but not with the results derived from stem–loop RT-qPCR [50].

## 5. Conclusions

The selection of reference genes is critically important for the accurate quantification of target gene expression in RT-qPCR analysis. To identify appropriate miRNA reference genes for normalizing the level of miRNA expression in *C. sinensis* and *C. reticulata* infected with a citrus canker pathogen, five algorithms: Delta Ct, geNorm, NormFinder, BestKeeper and RefFinder were used for screening reference genes. Poly(A) extension RT-qPCR and stem–loop RT-qPCR were performed to determine the most appropriate method for detecting expression patterns of miRNA. Our results showed that poly(A) RT-qPCR is a more reliable method than stem–loop RT-qPCR, and miR162-3p and miR472 genes in poly(A) RT-qPCR are the most stable internal control genes for miRNA profiling analysis of citrus infected by *Xcc*. 

## Figures and Tables

**Table 1:** Selected candidate reference gene primers and their parameters derived from RT-qPCR analysis

Gene Name	Forward Primer	Reverse Primer	Efficiency (E) (%)	*r*	Gene Type	Reference
*st-miR162-3p*	GCGGGCTCGATAAACCTCTGC	GTGCAGGGTCCGAGGT	104.7	0.9999	miRNA	
*st-miR166b*	AGCGGTCGGACCAGGCTTCAT	GTGCAGGGTCCGAGGT	87.51	0.9976	miRNA	[31]
*st-miR396a*	CGCGGCTTCCACAGCTTTCTT	GTGCAGGGTCCGAGGT	86.84	0.9988	miRNA	
*st-miR472*	CGCGGCTTTTCCCACACCTCC	GTGCAGGGTCCGAGGT	94.17	0.9975	miRNA	
*st-miR482b*	TCATCTCTTGCCCACCCCTCC	GTGCAGGGTCCGAGGT	99.57	0.998	miRNA	
*st-miR3954*	GCGGCGTGGACAGAGAAATCA	GTGCAGGGTCCGAGGT	87.73	0.9992	miRNA	
*st-miR403*	GTCGGCTTAGATTCACGCACA	GTGCAGGGTCCGAGGT	87.86	0.9965	miRNA	
*st-miR160*	GCGTCGGCCTGGCTCCCTGTA	GTGCAGGGTCCGAGGT	89.46	0.9962	miRNA	[30,67]
*csi-miR162-3p*	TCGATAAACCTCTGCATCCAG	GATCGCCCTTCTACGTCTAT	103.00	0.9730	miRNA	
*csi-miR166b*	TCGGACCAGGCTTCATTCCCGT	GATCGCCCTTCTACGTCTAT	102.51	0.9752	miRNA	
*csi-miR396a*	TTCCACAGCTTTCTTGAACTG	GATCGCCCTTCTACGTCTAT	99.31	0.9912	miRNA	
*csi-miR472*	TTTTCCCACACCTCCCATCCC	GATCGCCCTTCTACGTCTAT	99.30	0.9869	miRNA	
*csi-miR482b*	TCTTGCCCACCCCTCCCATTCC	GATCGCCCTTCTACGTCTAT	103.59	0.9918	miRNA	
*csi-miR3954*	TGGACAGAGAAATCACGGTCA	GATCGCCCTTCTACGTCTAT	99.73	0.9896	miRNA	
*csi-miR403*	TTAGATTCACGCACAAACTCG	GATCGCCCTTCTACGTCTAT	103.06	0.9968	miRNA	
*csi-miR160*	GCCTGGCTCCCTGTATGCCAT	GATCGCCCTTCTACGTCTAT	101.15	0.9878	miRNA	
*Csi-U4* *	GCAATGACGCAGCTTATGAGG	CAAAGGGAGCCCTTCCAGAA	88.64	0.9936	Non-coding RNA	[52]
*Csi-U5* *	GTGGGCACAGAGCGAACTAT	CGAAGAGAAACCCTCCAAAAA	89.57	0.9978	Non-coding RNA	[52]
*Csi-U6* *	ACAGAGAAGATTAGCATGGCC	GACCAATTCTCGATTTGTGCG	91.56	0.9999	Non-coding RNA	[52]
*Csi-snoR14*	TTCATGTCTGTCAATCCACTG	AACCTGTCGGGATTCAAGATA	93.25	0.9958	Non-coding RNA	[52]
*CsACT1*	ACGCTATCCTTCGTCTTG	GCTTCTCCTTCATATCCCT	102.25	0.9732	Protein coding RNA	[68]
*CsACT2*	AGTGCTTGATGGGTGAGTTC	GCAAGGAAGACGGTTGAGTA	96.29	0.9979	Protein coding RNA	[69]
*CsEF1α*	TGACTGTGCCGTCCTTATC	TCATCGTACCTAGCCTTTG	103.8	0.9796	Protein coding RNA	[68,70,71]
*CsGAPDH*	CCGTCTGCGATGTTCCACT	TCCAATGGGTCTCCGCTTCC	91.55	0.9972	Protein coding RNA	[72,73]
*CsPP2A*	TCGTAATCCGCAACCCTAT	ACGGCATCGTTTCACTCTAA	97.87	0.9942	Protein coding RNA	[74,75]
*CsTUA5*	CTATCTACCCTTCTCCTCAG	TTAGTGTAAGTTGGCCTCT	97.79	0.9917	Protein coding RNA	[76,77]
*CsTUB4*	ATCCCGCCTAAGGGTCTG	CTCGGTGAACTCCATCTCG	98.64	0.9923	Protein coding RNA	[78,79]
*CsUBC28*	GGTAGCATTTGCCTTGATA	GCAGTGACCTAACCCATT	103.65	0.9988	Protein coding RNA	[52,80]

* The primers were obtained from Kou et al., 2012 [52] and the reference referred to citrus homologous genes being screened as stable internal reference genes in other plants.

**Table 2:** Ranking of selected candidate genes based on the expression stability values calculated by Delta Ct, BestKeeper, NormFinder, geNorm and RefFinder in sweet orange (blue columns) and Ponkan (black columns).

Ranking	Delta Ct	BestKeeper	NormFinder	geNorm	RefFinder	Delta Ct	BestKeeper	Normfinder	geNorm	RefFinder
1	miR162-3p	st-miR472	miR162-3p	UBC28	miR162-3p	miR403	miR162-3p	miR403	miR162-3p	miR162-3p
2	miR472	miR160	miR472	miR166b	miR472	miR428b	miR472	miR428b	miR472	miR403
3	miR160	st-miR428b	miR160	miR472	miR160	miR162-3p	st-miR166b	miR160	miR428b	miR472
4	st-miR428b	miR166b	st-miR428b	miR162-3p	miR166b	miR160	miR160	miR162-3p	miR403	miR428b
5	st-miR472	miR472	st-miR472	miR160	st-miR472	st-miR472	miR166b	st-miR472	miR160	miR160
6	UBC28	miR428b	UBC28	miR428b	UBC28	miR472	miR403	UBC28	st-miR166b	st-miR472
7	miR166b	miR162-3p	miR166b	ACTIN2	st-miR428b	UBC28	miR428b	miR472	st-miR472	st-miR166b
8	miR428b	miR396a	miR428b	miR396a	miR428b	PP2A	st-miR472	PP2A	PP2A	UBC28
9	miR396a	snoR14	miR396a	st-miR472	miR396a	st-miR166b	UBC28	st-miR162-3p	UBC28	PP2A
10	ACTIN2	ACTIN2	ACTIN2	st-miR428b	ACTIN2	st-miR162-3p	PP2A	st-miR428b	st-miR403	miR166b
11	st-miR166b	st-miR166b	st-miR166b	miR403	st-miR166b	st-miR428b	st-miR403	st-miR166b	st-miR162-3p	st-miR162-3p
12	st-miR396a	UBC28	st-miR396a	U4	st-miR396a	st-miR403	st-miR160	st-miR403	st-miR428b	st-miR403
13	miR403	st-miR396a	miR403	st-miR166b	snoR14	miR396a	st-miR428b	miR396a	miR166b	st-miR428b
14	U4	U4	U4	st-miR396a	U4	miR166b	st-miR162-3p	miR166b	miR396a	miR396a
15	snoR14	miR3954	snoR14	snoR14	miR403	st-miR396a	miR396a	st-miR396a	st-miR396a	st-miR396a
16	PP2A	st-miR3954	PP2A	PP2A	EF1a	EF1a	EF1a	EF1a	st-miR160	EF1a
17	EF1a	EF1a	EF1a	EF1a	PP2A	ACTIN2	st-miR396a	ACTIN2	EF1a	st-miR160
18	TUB4	st-miR160	TUB4	st-miR160	st-miR160	TUA5	snoR14	TUA5	st-miR3954	ACTIN2
19	st-miR160	U6	st-miR160	TUB4	miR3954	st-miR160	ACTIN2	miR3954	miR3954	TUA5
20	miR3954	miR403	miR3954	miR3954	TUB4	miR3954	U6	st-miR160	ACTIN2	miR3954
21	st-miR403	PP2A	ACTIN1	st-miR403	st-miR3954	st-miR3954	TUA5	st-miR3954	TUA5	st-miR3954
22	st-miR3954	TUB4	st-miR403	st-miR3954	st-miR403	snoR14	U4	snoR14	U6	snoR14
23	ACTIN1	ACTIN1	st-miR3954	st-miR162-3p	ACTIN1	U4	st-miR3954	TUB4	U4	U6
24	st-miR162-3p	st-miR403	st-miR162-3p	ACTIN1	U6	TUB4	miR3954	U4	snoR14	U4
25	U6	st-miR162-3p	U6	U6	st-miR162-3p	U6	TUB4	U6	TUB4	TUB4
26	TUA5	TUA5	TUA5	TUA5	TUA5	ACTIN1	U5	ACTIN1	ACTIN1	ACTIN1
27	U5	U5	U5	U5	U5	U5	ACTIN1	U5	U5	U5
28	GAPDH	GAPDH	GAPDH	GAPDH	GAPDH	GAPDH	GAPDH	GAPDH	GAPDH	GAPDH

**Table 3:** Descriptive statistics of candidate genes based on BestKeeper in sweet orange.

	Geo Mean (Ct)	AR Mean (Ct)	Min (Ct)	Max (Ct)	Std dev (± Ct)	CV (% Ct)	Min (x-fold)	Max (x-fold)	Std. dev. (± x-fold)	Coeff. of corr. (*r)*	*p*-Value
U4	12.57	12.61	9.83	14.11	0.83	6.6	−6.66	2.92	1.78	0.669	0.005
U5	10.96	11.08	8.38	14.76	1.44	12.95	−5.97	13.96	2.7	0.555	0.026
U6	12.66	12.72	9.16	14.35	0.96	7.53	−11.29	3.23	1.94	0.271	0.309
snoR14	16.37	16.41	13.75	18.08	0.74	4.53	−6.16	3.27	1.67	0.58	0.019
ACTIN2	20.2	20.22	18.86	21.93	0.75	3.7	−2.54	3.31	1.68	0.447	0.083
ACTIN1	24.03	24.07	21.96	27.39	1.04	4.31	−4.19	10.29	2.05	0.556	0.025
UBC28	19.91	19.93	18.82	22.28	0.78	3.93	−2.12	5.18	1.72	0.769	0.001
TUA5	21.16	21.22	18.06	25.37	1.17	5.5	−8.6	18.45	2.25	0.575	0.02
EF1a	22.7	22.73	20.06	25.12	0.94	4.12	−6.24	5.35	1.91	0.575	0.02
TUB4	23.13	23.17	21.15	26.01	1.02	4.39	−3.94	7.36	2.02	0.632	0.009
GAPDH	20.23	20.33	18.11	25.3	1.74	8.57	−4.34	33.66	3.35	0.582	0.018
PP2A	23.44	23.47	21.71	27.04	0.97	4.14	−3.31	12.13	1.96	0.754	0.001
miR162-3p	18.17	18.19	17.18	19.18	0.67	3.68	−1.99	2.01	1.59	0.809	0.001
miR396a	13.53	13.56	11.7	15.1	0.73	5.35	−3.57	2.96	1.65	0.584	0.018
miR428b	14.71	14.73	13.13	16.71	0.66	4.5	−2.99	4	1.58	0.535	0.033
miR160	19.71	19.72	18.7	20.99	0.48	2.43	−2.01	2.43	1.39	0.621	0.01
miR403	17.04	17.08	15.36	19.28	0.97	5.69	−3.2	4.73	1.96	0.709	0.002
miR472	14.5	14.52	12.81	15.87	0.61	4.17	−3.23	2.58	1.52	0.685	0.003
miR166b	19.87	19.89	18.82	22.23	0.58	2.91	−2.07	5.13	1.49	0.668	0.005
miR3954	19.93	19.96	18.16	22.27	0.87	4.38	−3.41	5.06	1.83	0.249	0.352
st-miR472	22.55	22.55	21.61	23.75	0.42	1.86	−1.91	2.3	1.34	0.331	0.21
st-miR428b	25.41	25.42	24.01	27.08	0.51	2	−2.65	3.18	1.42	0.543	0.03
st-miR396a	20.34	20.36	18.96	22.38	0.81	3.99	−2.6	4.11	1.76	0.458	0.075
st-miR166b	23.78	23.8	22.79	25.58	0.75	3.15	−1.99	3.48	1.68	0.387	0.138
st-miR3954	27.93	27.95	25.65	30.44	0.92	3.28	−4.85	5.71	1.89	0.153	0.573
st-miR160	36.04	36.06	34.41	39.03	0.96	2.65	−3.09	7.95	1.94	0.495	0.051
st-miR162-3p	28.09	28.13	25.83	31.31	1.12	4	−4.79	9.32	2.18	0.438	0.089
st-miR403	23.02	23.06	21.61	26.97	1.04	4.52	−2.67	15.41	2.06	0.421	0.105

Geo mean (Ct): the geometric mean of Ct; AR mean (Ct): the arithmetic mean of Ct; Min (Ct) and Max (Ct): the extreme values of Ct; SD (± Ct): the standard deviation of the Ct; CV (%Ct): the coefficient of variance expressed as a percentage on the Ct level; Min (x-fold) and Max (x-fold): the extreme values of expression levels expressed as an absolute x-fold over- or under-regulation coefficient; SD (± x-fold): standard deviation of the absolute regulation coefficients.

**Table 4:** Descriptive statistics of candidate genes based on BestKeeper in Ponkan.

	Geo Mean (Ct)	AR Mean (Ct)	Min (Ct)	Max (Ct)	Std (± Ct)	CV (% Ct)	Min (x-fold)	Max (x-fold)	Std dev (± x-fold)	Coeff. of corr. (*r*)	*p*-Value
U4	12.33	12.39	9.77	14.05	0.95	7.67	−5.91	3.29	1.93	0.296	0.266
U5	11.22	11.33	8.9	14.24	1.31	11.55	−4.98	8.13	2.48	0.339	0.199
U6	13.1	13.14	11.08	14.87	0.89	6.75	−4.05	3.41	1.85	0.001	0.843
snoR14	16.34	16.39	12.43	18.09	0.85	5.21	−14.98	3.38	1.81	0.595	0.015
ACTIN2	19.6	19.63	17.85	22.9	0.86	4.36	−3.35	9.88	1.81	0.631	0.009
ACTIN1	24.38	24.44	21.91	28.04	1.53	6.26	−5.54	12.65	2.89	0.694	0.003
UBC28	19.81	19.83	17.01	20.56	0.5	2.52	−6.96	1.68	1.41	0.803	0.001
TUA5	21.42	21.46	18.22	24.45	0.9	4.18	−9.21	8.15	1.86	0.722	0.002
EF1a	23.08	23.11	20.77	25.58	0.79	3.44	−4.97	5.65	1.73	0.579	0.019
TUB4	23.06	23.12	19.95	26.8	1.11	4.8	−8.65	13.34	2.16	0.823	0.001
GAPDH	20.29	20.43	18.14	27.17	1.95	9.54	−4.43	117.99	3.86	0.506	0.046
PP2A	23.11	23.13	20.37	23.8	0.5	2.18	−6.69	1.61	1.42	0.706	0.002
miR162−3p	18.58	18.58	18.03	19.46	0.25	1.33	−1.46	1.84	1.19	0.415	0.11
miR396a	14.75	14.78	13.18	16.98	0.78	5.28	−2.97	4.7	1.72	0.698	0.003
miR428b	15.88	15.89	14.52	17.02	0.44	2.77	−2.56	2.21	1.36	0.687	0.003
miR160	20.49	20.49	19.31	21.16	0.42	2.07	−2.26	1.6	1.34	0.627	0.009
miR403	17.35	17.36	15.93	18.43	0.44	2.52	−2.68	2.11	1.35	0.851	0.001
miR472	15.37	15.37	14.92	16.06	0.26	1.66	−1.36	1.62	1.19	0.206	0.444
miR166b	20.23	20.23	19.09	21.29	0.43	2.15	−2.2	2.09	1.35	0.04	0.883
miR3954	19.57	19.61	17.6	21.07	1.04	5.29	−3.93	2.82	2.05	0.496	0.051
St-miR472	23.4	23.41	22.19	24.36	0.45	1.93	−2.31	1.94	1.37	0.54	0.031
St-miR428b	26.71	26.72	25.72	27.79	0.68	2.54	−1.98	2.12	1.6	0.43	0.097
st-miR396a	21.46	21.48	20.01	23.24	0.83	3.86	−2.73	3.43	1.78	0.389	0.136
st-miR166b	24.32	24.32	23.32	25.46	0.41	1.67	−2	2.21	1.33	0.18	0.504
st-miR3954	27.97	27.99	25.9	30	0.99	3.52	−4.18	4.09	1.98	0.315	0.235
st-miR160	36.87	36.88	34.82	38.74	0.65	1.77	−4.14	3.65	1.57	0.001	0.928
st-miR162-3p	28.92	28.93	26.95	30.16	0.7	2.44	−3.91	2.36	1.63	0.648	0.007
st-miR403	23.52	23.53	22.27	24.53	0.6	2.53	−2.38	2.01	1.51	0.348	0.187

**Table 5:** Gene expression stability of candidate reference genes calculated by RefFinder in sweet orange and Ponkan infected by *Xcc*.

Ranking	Sweet Orange		Ponkan		Protein Coding	
	Poly(A) RT	Stem-loop RT	Poly(A) RT	Stem-Loop RT	Sweet Orange	Ponkan
1	miR162-3p	st-miR396a	miR160	st-miR472	UBC28	UBC28
2	miR160	st-miR162-3p	miR162-3p	st-miR403	PP2A	PP2A
3	miR472	st-miR166b	miR472	st-miR162-3p	EF1a	EF1a
4	miR166b	st-miR472	miR403	st-miR166b	ACTIN2	TUA5
5	miR396a	st-miR428b	miR428b	st-miR160	ACTIN1	ACTIN2
6	miR428b	st-miR403	miR166b	st-miR428b	TUB4	TUB4
7	miR403	st-miR160	miR396a	st-miR396a	TUA5	ACTIN1
8	miR3954	st-miR3954	miR3954	st-miR3954	GAPDH	GAPDH

## Supplementary Materials

The following are available online at https://www.mdpi.com/2073-4425/11/1/17/s1, Table S1: Primer sequences used in stem-loop reverse transcription.
