# Supplementary material for: Identification of Appropriate Reference Genes for Normalizing miRNA Expression in Citrus Infected by Xanthomonas citri subsp. citri
Source: Genes (Basel). 2019 Dec 23;11(1):17. doi: 10.3390/genes11010017 (PMC7017248; doi:10.3390/genes11010017)
Supplement: Supplementary file 1 [file genes-11-00017-s001.pdf]

Table S1. Primer sequences used in stem-loop reverse transcription

miRNA	Stem-loop sequence
**miR472**	GTCGTATCCAGTGCAGGGTCCGAGGTATTCGCACTGGATACGAC**GGGATG**
**miR428b**	GTCGTATCCAGTGCAGGGTCCGAGGTATTCGCACTGGATACGAC**GGAATG**
**miR396a**	GTCGTATCCAGTGCAGGGTCCGAGGTATTCGCACTGGATACGAC**CAGTTC**
**miR166b**	GTCGTATCCAGTGCAGGGTCCGAGGTATTCGCACTGGATACGAC**ACGGGA**
**miR3954**	GTCGTATCCAGTGCAGGGTCCGAGGTATTCGCACTGGATACGAC**TGACCG**
**miR160**	GTCGTATCCAGTGCAGGGTCCGAGGTATTCGCACTGGATACGAC**ATGGCA**
**miR162-3p**	GTCGTATCCAGTGCAGGGTCCGAGGTATTCGCACTGGATACGAC**CTGGAT**
**miR403**	GTCGTATCCAGTGCAGGGTCCGAGGTATTCGCACTGGATACGAC**CGAGTT**

Note： The bold codes are the sequence that bind with mature miRNA.
